# Can I Phone a Friend? Exploring the Use of Digital Devices in Clinical Exams

**DOI:** 10.1111/tct.70007

**Published:** 2024-12-22

**Authors:** Hannah Gillespie, Helen Reid, Kathy Cullen

**Affiliations:** ^1^ School of Medicine Newcastle University Newcastle upon Tyne UK; ^2^ Centre for Medical Education Queen's University Belfast Belfast UK

**Keywords:** assessment, medical education, Objective Structured Clinical Examinations

## Abstract

**Background:**

Objective structured clinical examinations (OSCEs) are used globally to assess health professional learners' clinical skills and applied knowledge. Despite innovations with simulated participants, manikin technology and real patient involvement, there remains a gap between ‘real‐life’ practice and ‘OSCE experience’. For example, although mobile phone use is increasingly common in clinical practice; however, it would represent a significant disruption to established assessment practices in OSCEs. We aimed to explore student use of mobile phones during OSCE assessment, with a focus on exam security, equity and relevance to practice.

**Methods:**

Cultural Historical Activity Theory (CHAT) allows us to conceptualise and analyse complex systems such as those of OSCE assessment. We recruited a range of stakeholders in a UK medical school setting to participate in focus group interviews exploring our stated aim. Transcripts were analysed using CHAT as a theoretical lens to construct an activity system of assessment and identify emerging tensions around the use of a potentially disruptive technology: students' own mobile phones.

**Findings:**

Seven examiners, 13 medical students, and two simulated participants participated in three focus groups. Three sources of tension were identified: between the tools of assessment and practice, of exam security and exam relevance, and of medical students as people and professionals.

**Discussion and Conclusion:**

This study exemplifies how a seemingly small disruption in a complex system (introducing a mobile phone—a tool used in everyday practice) can help us understand and describe the unwritten rules of assessment.

## 1 Background

First described in 1975 [1], Objective Structured Clinical Examinations (OSCEs) are now used globally to assess health professional students' clinical skills and knowledge. OSCEs are designed to assess a range of procedural, examination and communication skills. Simulated situations, mark schemes and patients are used so that many students can be reliably assessed by a pool of examiners. Within undergraduate medical education, OSCEs are ‘high‐stakes’, often marking critical transition points for students before graduation.

Educators hope to prepare students to be capable clinicians. We know, however, that students often feel unprepared to practise upon graduation [2, 3, 4, 5]. Much of their learning has been focused on demonstrating competency in high‐stakes examinations, including OSCEs [6]. There is a significant gap between ‘real‐life’ clinical practice and the ‘OSCE experience’, which creates tensions for both student and examiner [7]. One marked difference is the ‘tools’ that students have available in an exam situation. In clinical practice, students are encouraged to use all available resources to help them learn and care for patients. These resources are often not available during assessment—and, if provided, may only be available in unfamiliar forms. For example, the British National Formulary (a pharmaceutical reference) may be provided as a book or print‐out, rather than the mobile phone application (app) so ubiquitous in authentic clinical practice.

Educators have sought to bring examinations, in content and environment, into closer alignment [8]. Methods used to make OSCEs more ‘real’ include special effects such as moulage and simulating a believable surrounding [9]. Others have championed the inclusion of ‘real’ as well as simulated participants [10]. However, innovations in OSCEs can be limited by logistics, cost and concerns about exam security and standardisation. Here, we seek to explore how we might enable students to use a familiar resource—a mobile phone, which although a common tool in clinical practice, is often prohibited during an OSCE.

Activity theory (AT) gives researchers a lens to understand complex systems. AT analyses ‘activity systems’, which are object‐oriented, collective and culturally mediated human activity [11]. We regarded mobile phones as a ‘tool’ that clinicians and students use in the Activity System of Clinical Practice. These devices provide clinicians with ‘convenient and rapid access to evidence‐based information’. [12] Clinicians use mobile phones in all areas of practice: Previous studies highlight the widespread ownership of mobile phones amongst healthcare staff, with reported subjective benefits to clinical practice [13]. The increasing use and availability of mobile phones in the clinical environment have led to the development of specific apps to enhance communication between clinicians, improve learning, support clinical decision making and access health records.

We set out to understand how mobile phones could be introduced to assessment. In doing so, we had two aims: first, practically, to understand anticipated opportunities and challenges from students, simulated patients and examiners, and, second, as introducing a new tool had the potential to make longstanding tensions visible, we aimed to develop our understanding of tensions between the Activity Systems of Assessment and Practice, which we could then use as opportunities for expansion. Our research questions were as follows:

1. How do students, examiners and simulated patients view the use of mobile phones within OSCEs?
2. What tensions are exposed through the proposed implementation of a new tool within OSCEs?

## 2 Methods

### 2.1 Setting

The medical programme is a 5‐year undergraduate programme, in which students spend time in a range of clinical placements in hospital and primary care. As is common in many medical schools globally, students undertake a multistation OSCE each year, which assesses a range of specialities and skills. Students' progression through the programme is dependent on successful performance in these examinations. These summative OSCEs were the focus of the study. Clinicians, educators and professional support staff work together to organise the administration and practicalities of the assessment. In addition, both senior and junior clinicians from primary and secondary care are trained to act as examiners. Members of the public, who have undergone specific training for the role, act as simulated participants during the examination.

### 2.2 Conceptual Orientation

From an AT perspective, clinical education and assessment are activities, each of which is a composite of multiple actions [11]. Any activity is orientated towards an object: an external reality or problem space. AT attends to how speech and other symbols (clinical language and abbreviations), material tools (scrubs, stethoscopes and mark schemes) and rules (social interactions, codes of behaviour and skill‐mix) influence the complex links between subjects (doctors and students), the object (caring for patients and passing assessments) and outcomes of their object‐oriented activities. Tensions are of particular interest, as although these can be troublesome and disabling in the moment, they are also signposts towards new opportunities. By identifying tensions and analysing them, AT allows researchers in complex systems to identify opportunities to move towards better performance [14, 15].

### 2.3 Methodology

Consistent with our AT orientation, we took the activity system of assessment as our unit of analysis. We aimed to identify tensions, which could be addressed to understand how a new ‘tool’ might create opportunities or counterproductive tensions. We chose a dialogic qualitative methodology, stemming from the work of Bakhtin [16]. According to his dialogism theory, utterances (actions mediated by speech) mediate incessant reconstruction of activity systems, and participants' speech acts derive from their experiences in the sociocultural world of education and practice, rather than being solely artefacts generated by research settings. This theoretically grounded approach enabled us to collect data in the form of participants' words and analyse them in a way which developed our understanding of tensions within this activity system.

### 2.4 Recruitment

Recruitment took place during the assessment period of the 2021–2022 academic year. All fourth‐year medical students were contacted via email to inform them about the study. Two researchers (HG + KC) subsequently invited those who were willing to join in a focus group following a summative OSCE, undertaken as part of the usual curriculum, to participate. Similarly, simulated participants and examiners who were participating in the assessment but were not scheduled to be examining/participating at a particular time were invited to join a focus group taking place concurrently with an OSCE. The inclusion criteria were intentionally broad:

- To be a fourth‐year medical student, simulated participant or trained OSCE Examiner.
- Have participated in at least one previous OSCE in this role.
- Be willing to participate.

### 2.5 Data Collection

**FIGURE 1:**
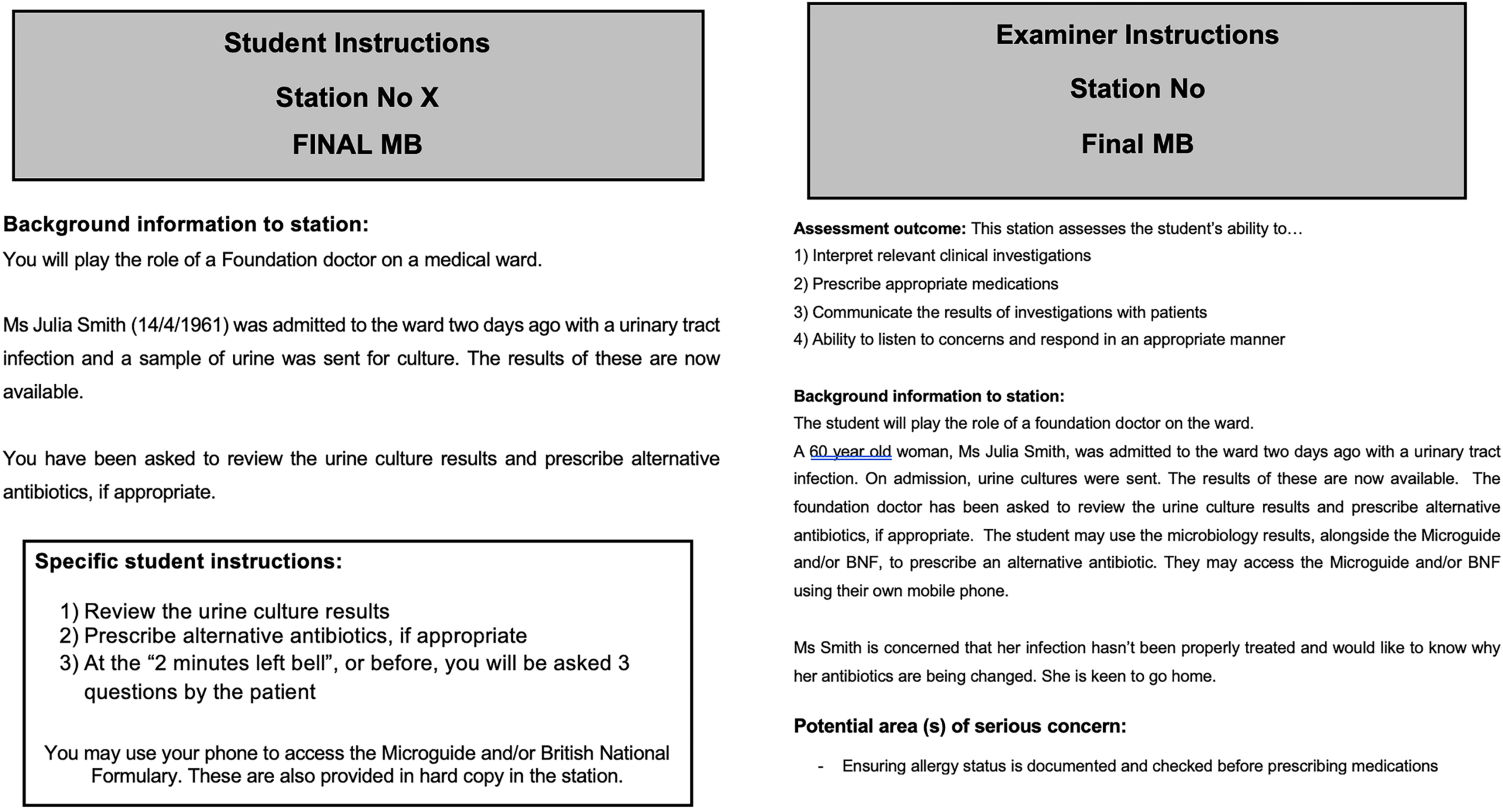
Discussion prompts.

HG and KC conducted three semistructured focus group interviews: one with fourth‐year medical students, one with simulated participants and one with examiners (who were both junior and senior clinicians). Focus groups were semistructured. Participants were briefed about the purpose of the study and offered the opportunity to ask questions. They were then invited to share any initial thoughts they may have had. After discussion around the topic of interest, participants were invited to review mock documents for an OSCE station, which had been designed for the purposes of the study. These documents acted as a stimulus for further discussion. They included mock instructions for candidates and examiners, briefing notes for simulated patients and a mark scheme. A condensed version of this prompt material is shown in Figure 1. Focus group interviews lasted between 45 min and 1 h 15 min. An interview guide is shown in Box 1.

Box 1 **Interview guide.**

1. Introductions of both researchers and participants.

2. Introduction to the study. Opportunity to share initial thoughts—could we use mobile phones effectively in clinical examinations?

3. Sharing the proposed template (Appendix 5: Mock OSCE Station).

4. Inviting comments on proposed template—what would work here? What would not work here? Do you think this is an improvement on previous OSCEs you have participated in? If so, how? If not, why? How have we made it worse?

5. What concerns would you have as a student/examiner/simulated patient/assessment lead? We were concerned that some students/examiners/patients would be worried about cheating/professionalism/equity—what do you think of that?

6. How might we change this scenario it to make it more acceptable?

7. Are there any other improvements that come to mind that would help us make OSCEs more lifelike?

### 2.6 Data Analysis

Each interview was recorded and transcribed verbatim by HG. HG then searched the transcripts for informative dialogue relating to tensions in the form of participants' concerns, worries or suggestions for change. AT‐oriented analyses often focus on tensions due to the rich explanatory opportunities this analysis provides [8]. These sections of text were coded. We used inductive coding to group codes together into broader potential themes. HG, HR and KC then reread the transcripts in their entirety. They discussed their evolving interpretation via multiple synchronous and asynchronous research meetings. The themes were discussed and adapted iteratively throughout the interpretive process.

### 2.7 Ethical Approval

The Faculty of Medicine, Health and Life Sciences Research Ethics Committee approved the study (MHLS 21_131).

## 3 Findings

Seven examiners, 13 medical students and two simulated participants participated in three focus groups. A pseudonymised list of participants is available in Table 1. We present some practical considerations for educators in the discussion. For educators who seek to create assessment procedures that reflect clinical practice, we offer some practical tips for including digital technologies in OSCEs in Table 2. These include giving stakeholders sufficient time to prepare for any change, to establish clear guidance on what is being assessed and consider the type of device, and some considerations for ensuring exam security are maintained.

**TABLE 1:** Table of participants.

1	Examiner	M	Medical Consultant and Clinical Academic
2	Examiner	F	Medical Consultant
3	Examiner	M	Medical Consultant and Clinical Academic
4	Examiner	M	Anaesthetic Registrar
5	Examiner	F	Medical Registrar
6	Examiner	M	General Practice Registrar
7	Examiner	F	Clinical Fellow
8	Simulated Patient 1	M	Professional Actor
9	Simulated Patient 2	F	Academic and Artist
12	Student 1	F	4th year medical student
13	Student 2	F	4th year medical student
14	Student 3	F	4th year medical student
15	Student 4	F	4th year medical student
16	Student 5	F	4th year medical student
17	Student 6	F	4th year medical student
18	Student 7	F	4th year medical student
19	Student 8	M	4th year medical student
20	Student 9	F	4th year medical student
21	Student 10	M	4th year medical student
22	Student 11	F	4th year medical student
23	Student 12	M	4th year medical student
24	Student13	M	4th year medical student

**TABLE 2:** Practical suggestions for educators.

Give students time to prepare for potential changes in examination practices	If new digital devices are incorporated, this might require training in specific type of device being used and in specific applications being used. Sufficient notice of potential changes is required, for both students and examiners.	‘They might need to be some sort of training even on how to look them up on the app on the BNF and how to use the microguide, like a session for the students?’ (Ex4) ‘I think if you are consistently using it, you know, every time you go to CSEC (Clinical Skills Education Centre) from first year … you know that the iPad is for CSEC stuff and you'll get to learn how to use it. You have a full year before your exams come up to work it out’ (MS7)
2. Establish clear guidelines about what is being tested	Students and examiners sought clear guidelines that devices were an adjunct to assessment, rather than the focus of the assessment itself. It should not be testing students on their familiarity or ability to use the device.	‘I think if you just had it like when we go for our skills that week before the exams and there's an iGel (airway adjunct) and there's a LMA (airway adjunct) sitting, you have the iPad sitting as well and you can learn how to use it as long as it was made clear to students, you know, it's to be used, not like asked a question on at the end, not going to be a full station where you are setting up an iPad, so it's not like a test of using an iPad. It's like an adjunct’. (MS12)
3. Think about the type of device	Although students, examiners and simulated participants all seen value in integrating new technology, they shared concerns about how this might be practically implemented. Using personal devices was complicated by concerns about exam security and equity. Having a standard university device, available to all students, was the most acceptable option. As all technology has the potential to fail, they suggested having readily available non‐digital back‐ups available.	‘Is it your mobile phone or is it a mobile phone?’ (MS2) ‘You would though give them the option, you'd say you feel free to use your own phone but here's a book here and an iPad here, yeah. However you find it most easy to get the information’. (MS4) ‘Yeah, and for me, like when you are just starting out with the iPad thing like having paper there too, just in case there's any issues because that would be a lot of stress when you are not used to it’. (MS11)
4. Exam security	Students and examiners were concerned about potential threats to exam security. There were potential solutions offered—such as using ‘flight mode’—however, the risks of exam security seemed to be a potential stumbling block to personal devices, and favoured university devices.	‘I guess with bringing your phone in during the actual OSCE, well will it be taken off us during the rest station and stuff like that? Cause anyone can look up stuff if no one's around them’. (MS8)
5. Potential new avenues	Throughout each interview, our participants thought about other potential opportunities integrating technology may provide. We share these here as potential inspiration for other educators.	Prescribing—using an electronic British National Formulary Calculations—such as calculating a patient's creatinine clearance Assessing risk—such as calculating a Well's score Accessing information on an electronic care record Requesting investigations, or discussing the results of these for patients Phoning for senior advice/assistance Following emergency algorithms

Our AT‐informed analysis of the data identified three sources of tension: the tension between the tools of assessment and practice, of exam security and exam relevance and of medical students as people and professionals.

### 3.1 Tension Between the Tools of Assessment and the Tools of Practice

Participants described a gap between education and practice: ‘you do hear again and again anecdotally about the disconnect between what people actually do as F1s and how university prepared them’ (examiner [Ex] 3). Aspects of assessment, such as strict time pressures, created artificial pressures, which widened this gap: ‘OSCEs don't give us enough time to do everything properly. Like, to do it as if we would do if we're on the ward, it's sort of like a half version of it sometimes’ (medical student [MS] 1).

Adding equipment, be that medical equipment or other props, was viewed to be a potential source of stress for students: ‘that's the thing with all of the, I call them the toys. Whenever the there's equipment in the room like, oh my god, like you see the stress they go through …’ (simulated patient [SP] 2).

OSCEs were expected to be simulated. Unlike in clinical practice, where students have access to their own resources, students and examiners expected that the same tools would be available to each student in exam settings. Introducing mobile phones, which could be from different brands and different network providers and have infinite combinations of downloaded apps, challenged examiners expectations around standardisation: ‘We would need to then have, well there are lots of different calculator apps and things, so we would need to have a [university] approved, simulated app—“we recommend this”’ (Ex1).

Participants highlighted how doctors in clinical practice are expected use information: ‘your job as a clinician is going to be to make the absolute best use of all the resources available to you’ (SP1). This ‘on the ground’ reality was removed from/in OSCE practices. Having access to relevant guidelines might better reflect clinical practice and open up new possibilities for learning: ‘I would have thought it opens up the potential for bigger learning. If you have that access all the time because presumably if you're having to just retain everything in your head then you haven't got time to interrogate the subject’ (SP 2). This had the potential to reduce the emotional burden of assessment: ‘that's your gold star for putting this in, is that it will relax them from having to be a font of every medical ailment, measurement, dosage that ever existed. And when they don't have to worry about that, what can they not turn their heads to?’ (SP2).

### 3.2 Exam Security and Exam Relevance

Student participants were particularly concerned about exam security and the association with using a mobile phone and cheating. They questioned, ‘do you actually trust us?’ (MS7). Using mobile phones was at odds with usual exam practices, and potential introduction introduced perceived opportunities for students to ‘cheat’. Students were used to using their mobile phones during day‐to‐day clinical practice but realised this would not be acceptable during assessment. They worried the almost *automatic* use of a mobile phone might be interpreted as cheating. Again, something accepted within authentic clinical practice was seen as problematic within an OSCE context: ‘I think just because we use our phones and we kind of have this sort of autopilot mode, so I think some people might not intentionally try to, but because I have a phone and I might just, you know, (gestures taking it out and using it)’. (MS13).

Participants had explored that ‘exam conditions’ in OSCEs were at odds with how clinicians actually practise. Adapting available technology had the potential to shift this. By allowing students to have access to guidelines and policies, exam relevance could be enhanced: ‘… examining closer to real life, because in real life you won't know off the top of your head what the local policy is’ (MS4).

Importantly, examiners spoke of the need to ensure the assessment was assessing overall skills, rather than just using a mobile phone: ‘It's important that the candidates understand that this [using the mobile phone] is not the full station’ (Ex4) and that ‘inclusion of the device was testing students' ability to access accurate information, rather than navigate the technology itself’ (Ex2). Finding the information was important but needed to be supplemented by wider skills in interpretation and explanation: ‘the poor candidate could Google all day and get all the information that they need, they're still not going to come across as a strong person or someone that you would readily and comfortably go back to’ (SP1).

### 3.3 Medical Students as People and Professionals

Students were expected to assume a professional role, and using personal devices was seen to potentially challenge this. Simulated participants felt this ‘personal’ device may blur ‘the professional barrier or boundary’ (SP2). In contrast, ‘an iPad, I think there's already inherently an air of professionalism with it because it's used more often as a tool, than it's used for anything personal’ (SP1).

The simulated processes within OSCEs—where students wore university issued scrubs (uniform)—were seen to enhance equity. Allowing students to bring in a mobile phone, which was more personal, could be potentially discriminate between candidates:

I don't think it would be necessarily very fair for people to bring in their own phones as well, because then you're like a lot of people have different models of phones, iPhones, Android, different speeds like battery, things like that. Like people could complain that kind of could discriminate. (MS9)

Participants felt there was difference between ‘your mobile phone’ and ‘a mobile phone’ (MS2). A generic device, which remained within the station, may be preferable, as personal mobile phones felt like “’a foreign object, a personal foreign object’ (SP2).

## 4 Discussion

In this study, students, examiners and simulated patients explored how mobile devices might be integrated within OSCEs, to align assessment practices with students' clinical experiences. In doing so, we have developed practical recommendations for educators. We have shown how this potential change in the activity exposed tensions between the tools of assessment and the tools of practice, between exam security and exam relevance and between medical students as people and professionals.

This study shows how a seemingly small change in a complex system can help us understand and describe the unwritten rules of the activity. It shows how these tensions can be exposed, which can subsequently be used as a platform for potential change. It also acts as an opportunity for educators, demonstrating how discussions with stakeholders could enable coproduction of educational innovations and explore potential consequences of their implementation.

Educators have grappled with the issue of exam security for some time [17]. OSCEs are an organisational challenge and are often impossible for all students to sit the exam at the same time [18]. Educators have sought to deal with issues of exam security by quarantining students [19] (as was routine practice at our university) or, conversely, intentionally/deliberately sharing some exam details beforehand to all students [20, 21]. This study has shown that exam security remains a relevant issue for students, examiners and simulated patients. Potential innovations may align assessment closer with practice but, in doing so, could compromise potential exam security.

Participants described how standardisation and reliability may also be threatened when educators seek to align OSCEs with clinical practice. The ability to test knowledge and skills in a simulated and reliable way has been thought of as a driving force in the widespread adoption of OSCEs [1, 7]. OSCEs, by definition, seek to standardise assessment processes. Others have described three types of work surrounding OSCEs—standardising work, defensibility work and accountability work [22]. Introducing mobile devices, which meant students had different tools from each other, challenged participants who had come to expect assessment to be reliable through standardisation. It could, however, help educators gear assessment towards the ‘realities of ever‐evolving work settings’. [23].

### 4.1 Limitations

This study has limitations. First, it was conducted in one university in the UK. Although each university has unique assessment processes, which may limit the transferability of these findings, the core features of OSCEs are likely similar between institutions. Second, all individuals in the study were actively involved in assessment. Although this allowed participants to reflect on their experiences in assessment and clinical practice, it may be that other clinicians (who are not educators or examiners) and patients (who do not act as simulated participants) could have offered a unique perspective. Third, our recruitment strategy meant the proportion of female participants is slightly higher than our overall cohort. We did not collect further demographic details from participants, but it is possible this group of participants is not reflective of our wider student population. Finally, although we hope focus groups are a strength of this work, it is possible that for some participants, this format may have made it harder to share their thoughts. We conducted different focus groups for students, examiners and simulated patients to minimise potential power differentials between these groups. Although every attempt was made to ensure participants felt comfortable sharing their experiences, it is still possible that power imbalances between the interviewers (including KC: Director of Assessment) might have prevented some participants sharing their views freely.

### 4.2 Implications

First, this study has a practical implication. For educators who seek to increase the ‘realism’ of their assessment procedures, we offer some practical tips for including digital technologies in OSCEs in Table 2. In our institution, we have used this study to influence change in our own assessment practices by introducing electronic resources on a generic tablet device. At present, we have not planned to integrate personal mobile phones during assessments. Second, this study has explored tensions between assessment and clinical practice. By discussing potential change, we have brought preexisting tensions to the fore. Educators should not necessarily seek to resolve these tensions but, rather, consider how they can be productively harnessed to bring about change within their own educational practices.

## 5 Conclusion

The ability to innovate and create novel assessment methods, which reflect clinical practice, may be challenged by the drive for educators to ensure OSCEs both simulated and reliable. This study has shown how one such attempt at innovating within assessment revealed tensions between the tools of assessment and the tools of practice, between exam security and exam relevance and between medical students as people and professionals.

### Author Contributions

**Hannah Gillespie:** investigation, writing – original draft, writing – review and editing, formal analysis, funding acquisition. **Helen Reid:** investigation, funding acquisition, writing – original draft, methodology, writing – review and editing, formal analysis, supervision. **Kathy Cullen:** conceptualization, investigation, funding acquisition, writing – original draft, methodology, writing – review and editing, formal analysis, supervision.

## Conflicts of Interest

The authors declare no conflicts of interest.

## Data Availability

The data that support the findings of this study are available from the corresponding author upon reasonable request.
